# Comparative Evaluation between Diameter Difference of the Thumb and Asymmetry of Lateral Cerebral Ventricles in Children with Developmental Delay: A New Finding

**Published:** 2015

**Authors:** Zarintaj KEIHANI DOUST, Mamak SHARIAT, Elham RAHIMIAN, Fatemeh TEHRANI, Gholamreza SADDIGHI

**Affiliations:** 1 Pediatric Neurologist, Department of pediatric neurology, Imam Khomeini Medical Complex, Tehran University of Medical Sciences, Bagher Khan Ave, Tehran, Iran.; 2 Maternal & Child Health Specialist, Maternal Fetal Neonatal Research Center, Tehran University of Medical Sciences, Vali-Asr, Hospital, Bagher Khan Ave, Tehran, Iran; 3 Radiologist, Tehran University of Medical Sciences. Vali- Asr Hospital, Bagher Khan Ave. Tehran, Iran.; 4 General Practitioner, Tehran University of Medical Sciences. Vali- Asr Hospital, Bagher Khan Ave. Tehran, Iran.; 5 Pediatrician, Tehran University of Medical Sciences. Vali- Asr Hospital, Bagher Khan Ave. Tehran, Iran.

**Keywords:** Motor developmental delay, Asymmetrical thumbnail size, Asymmetry of lateral ventricles

## Abstract

**Objective:**

Anthropometry (measurement of body dimensions) has been used for clinical diagnosis of growth and developmental disorders during pregnancy and after birth. Different brain volumes have also been shown in abnormal developmental disorders. This study compares the different horizontal diameters of the left- and right-hand thumbnails and asymmetry of lateral cerebral ventricles in children with developmental delays.

**Materials & Methods:**

This retrospective case control study was carried out in the Pediatric Neurologic Outpatient of a university hospital in Tehran, Iran (2009–2011). Twenty-eight patients with motor developmental disorders (case) and 28 healthy individuals (control) had brain MRIs and volume of lateral cerebral ventricles size had been studied. The maximum horizontal diameters of the left and right thumbnails were measured by calipers during physical and neurological exams by a pediatric neurologist. Finally, we compared and analyzed different horizontal diameters of the left and right hand thumbnails and asymmetry of lateral cerebral ventricles.

**Results:**

There was a significant correlation between asymmetry of brain lateral ventricles size and mean difference of horizontal diameter of thumb nails (P = 0.0001). A meaningful relation between brain hemispheres asymmetry and developmental delay (P = 0.04) was seen.

**Conclusion:**

The asymmetry of thumbnails can be a marker for asymmetry of lateral ventricles and child developmental delays.

## Introduction

The prevalence of developmental delay is reported about 1–3% in children and ventricle abnormality is a common feature with this complication (1,2). Anthropometry (measurement of body dimensions) has been used for clinical diagnosis of growth disorders during pregnancy and after birth. The disproportion of head circumference and child height or asymmetry in brain and skull diameters can demonstrate genetic, metabolic, fetal developmental disorders, mental retardation, and brain tumors (3). Some studies have shown different lateral ventricles size (left and right side) in different ages and genders (4). Different brain volumes have been indicated in preterm infants with abnormal developmental outcome (5). There is a significant relation between growth and skull size (6). The knowledge about the size of dominant cerebral hemispheres had been of interest every time for medical teams due to the influence of diagnosis and therapy measures. Ventricular brain size can determine the development of the brain (7). Developmental disorder is incurable but the problem is immutable and timely detection of movement patterns during childhood may modify the treatment and rehabilitation (8). In the past, most studies have examined limb movement and its relation to other brain volumes (3, 6, 8), but there is no study about the size of thumbnails and lateral ventricular size. Each criterion related to the brain ventricles dimension may help with neurodevelopmental disorder prediction, early diagnosis, and treatment. This study compares different horizontal diameter of the leftand right-hand thumbnails as a marker of asymmetry of lateral ventricles.

## Materials & Methods

This is a retrospective, case control study that compares 1–5 year old children horizontal diameter of right- and left-hand thumbnail size and lateral cerebral ventricular sizes in children along with brain imaging at the Pediatric Neurology Outpatient of Imam Khomeini Teaching Hospital (Tehran, Iran) in 2009–2011. In twenty-eight patients with motor developmental delay (cases) and 28 healthy individuals, brain MRIs have been done for other reasons (control) and were introduced in this study. Inclusion criteria included significant differences in the diameter of the right and left nail in children with developmental delays and in the control individuals. Children with other neurological or anatomical problems that can interfere with the validity of the study, such as hemiplegic cerebral palsy, Autism, porencephalic cyst, and arterio-venous malformation (AVM) were excluded. This study was approved by Ethic Committee of Tehran University of Medical Science. All parents of participants gave informed consent. There were no additional costs for those who gave informed consent. A pediatric neurologist after general and neurological examination measured the maximum horizontal diameter of left and right thumbnails with calipers in millimeters. For estimating the lateral ventricles diameters, we did brain MRIs without contrast at our center and then sent all images to Germany for volumetric measurement. MRIs were acquired from a 3 Tesla Siemens Trio Scanner (Siemens, Erlangen, Germany) using 8 channel head coil and body coil transmitter. Further the image protocol included 3-D, T1 weighted MPRAGE sequence with imaging para tTR, TE, Flip, 17uo, 3.3, 15,256 mm, field 160 slices, and 1mm by 1mm cubic voxel dementia pixel band width 149 HZ. Researcher used a telephone and followed-up with the participants and no one was excluded. Data was collected by questionnaire and recorded on a form for all individuals. All data were statistically analyzed by the SPSS (ver 18) with T test, Chi-Square, and Pearson Correlation tests. P-value lesser than 0.05 was considered significant. The power of the study was 80%.

## Results

Twenty-eight healthy individuals and 28 patients with motor developmental delay were assessed. The target population was 1–5 year old children with 10 (35.7%) girls and 18 boys (64.3%) in each group. Table(s) 1 and 2 show the mean diameter of thumbnails, the difference size of lateral ventricles, and their relation with child developmental delays. Our study showed a significant relation between frequency of diameter difference of thumbnails in each group (P = 0.01). In addition, our findings showed a significant correlation between frequency of diameter difference of thumbnails and asymmetry of brain lateral ventricles size (r = -.56, P-value = 0001). Children with developmental impairment had a greater right-left thumbnail difference (P = 0.01), smaller right (P = 0.91), and left ventricles (P = 0.04), and larger difference of lateral ventricles size (0.04). On the other hand, based on our results, no correlation was seen between the mean diameter difference of thumbnails and sex and age. 

**Table1:** Frequency of Difference of Horizontal Diameter of Right and Left Thumbnail in Each Group

Patient groups	with motor developmental delay	normal	P-value
Difference of horizontal diameter of right & left thumbnails N (%)	18(64%)	10(36%)	0.01

**Table 2:** Lateral Ventricles Size and Their Difference in Each Group

Patient groups	with motor developmental delay	normal	P-value
Right lateral ventricles(mm^3^) (mean ± SD)	204.25 ± 44.67	205.32 ± 59.84	0.91
Left lateral ventricles(mm3) (mean ± SD)	187.64±40.69	213.07±49.92	0.04
Difference of lateral ventricle size	16.61±3.98	7.65±10.08	0.04

## Discussion

We noticed that children with developmental disorders had different thumbnail sizes and an asymmetry of lateral ventricles. Based on our results, differences in right and left thumbnails may significantly predict asymmetry in brain ventricles. We concluded that when the left nail is smaller than right nail, the right cerebral ventricle is larger. Different diameters of thumbnails could be a marker of asymmetry of lateral ventricles or at least a warning tool for physicians to do more investigation. Although developmental disorders are incurable, early diagnosis can effect on rehabilitation and medical therapeutic interventions. The first notice on dominant asymmetry of brain hemispheres was reported in 1865 (9,13). There are many causes that effect ventricular size in children with neuro-developmental delays, such as brain atrophy, post hemorrhagic dilatation, hydrocephalus, extensive lesions, and decreased in early cranial growth due to neonatal illness (10). Shape and transverse nail curvature are influenced by gender, age, and hand dominance. In addition, difference in the shape and size of nail plates can be a symptom of local, systemic, or genetic disorders (11). For instance, incontinentia pigmenti (IP), a genetic disorder (resulting developmental delays) may effect nail shape and size by dystrophic changes (12). Different ventricular brain size in these children was reported in some studies as follows. Cooke et al. showed that of VLBW infants who had been examined at the ages of 12 and 13 years with an MRI, there were 24 cases who had asymmetric ventricular dilatation (13). Leisti et al. also reported that among 15 children with mental retardation, 4 cases had symmetrical or unilateral ventricular enlargement and 5 children had large temporal horns with symmetric or asymmetric ventricle widening (14). Another study from the Netherlands showed that of 80 children 5.3 + 4.1 years old with abnormal neurological signs, there were 11 cases that had asymmetry in their ventricles with the anomaly were more common on the left side (1). Our study also showed a significant relation between mean diameter difference of thumbnails and mean lateral ventricular size. We supposed that some complications like hypoxic lesions, intra ventricular hemorrhage, compressions, and other abnormalities in the brain hemispheres, especially during the developmental stage cause some body anthropometric changes. However, many studies assessed the correlation between the dominancy of motor organs and brain ventricles. A few studies have evaluated the relation between anatomical aspects of the extremities particularly thumbnail size and hemispheres ventricles. Brain volumetric assessment in childhood developmental delayed children was not done as a usual procedure anywhere worldwide. We suggest measurement of brain ventricle volumes in these complicated children should be considered. We found the dimension of right and left thumbnails may be applied as a useful clinical diagnosis tool to provide important insights into neurodevelopmental diseases as well. Our study limitations include a small sample size and the need for transmission of brain imaging to another center for volumetric measurements. 

## Notes

**Acknowledgement:** This retrospective case control study was carried out in the Pediatric Neurologic Outpatient of a university hospital Tehran- Iran (2009- 2011).
